# Assessing the Impact of Helicobacter pylori Infection and Inflammatory Bowel Disease on Pulse Wave Velocity and Arterial Stiffness

**DOI:** 10.7759/cureus.14944

**Published:** 2021-05-10

**Authors:** Lizbet Chavez, Harvey N Mayrovitz

**Affiliations:** 1 Medicine, Nova Southeastern University Dr. Kiran C. Patel College of Osteopathic Medicine, Davie, USA; 2 Medical Education, Nova Southeastern University Dr. Kiran C. Patel College of Allopathic Medicine, Davie, USA

**Keywords:** pulse wave velocity, inflammatory bowel disease, h. pylori, arterial stiffness, crohn’s disease, ulcerative colitis, anti-tnf-alpha

## Abstract

There is some evidence that pulse wave velocity (PWV) is increased in gastrointestinal conditions such as *Helicobacter pylori *(*H. pylori*) infections and inflammatory bowel disease (IBD). However, the limited number of well-designed studies and sometimes inconsistent results have yielded more questions than answers highlighting a need for further investigation. The purpose of this review is to clarify the effects of *H. pylori* infections and IBD on PWV and arterial wall stiffness. The goal is to highlight the extent of the linkage between these gastrointestinal conditions and PWV and to help evaluate the practicality of PWV as a potential clinical diagnostic aid when examining arterial stiffness. PubMed, CINAHL, EMBASE, Cochrane Central Register of Controlled Trials, and Biomedical Reference Collection: Comprehensive were used to search for the full-text English language articles using keywords “pulse wave velocity” combined with either “*H. pylori*” or “IBD” present anywhere in the abstracts. A total of 59 papers matched the search criteria and were retrieved for evaluation. These were screened based on their relevance and availability of published papers. Full papers were analyzed based on inclusion criteria with a total of 10 articles selected and included in this review. In younger populations, *H. pylori *seropositivity might play a role in the development of arterial stiffness, as assessed by PWV; while in older populations, the effect of *H. pylori *on arterial stiffness seems to be minimal, with aging playing a major role in these older patients. PWV does not appear to be an accurate parameter to assess arterial stiffness in older patients with *H. pylori*. On the other hand, PWV might be useful to assess the efficacy of anti-tumor necrosis factor-alpha (anti-TNF-alpha) immunotherapy in reducing the degree of arterial stiffness caused by inflammation in patients with IBD.

## Introduction and background

Pulse wave velocity (PWV) is a parameter used among biomedical professionals to assess arterial wall stiffness [1]. It represents the velocity at which pulses from the left ventricle move through central and peripheral blood vessels [1]. PWV is also regarded as a reliable tool to predict the risk of adverse cardiovascular events in diverse populations and disorders [2]. For example, PWV has been validated as a strong marker for future cardiovascular events in patients with hypertension, diabetes, and end-stage renal disease [2,3]. Although peripheral arteries, such as brachial and femoral, tend to be stiffer than central arteries, like the carotid and aorta, peripheral arteries are overall less susceptible to stiffening due to aging [1-3]. Nevertheless, the pulse wave velocity measured from the carotid to the femoral artery, which is a measure of the PWV in the aorta, is considered by some to be the gold standard to assess arterial stiffness [3]. 

PWV can be calculated as the path length traveled by the pulse divided by the time taken, either within a vessel (local) or between two segments (regional) for any vessels in the body [2]. Aortic pulse wave velocity is most accurately measured using pressure catheters, however, due to its invasive and complex nature, this method tends to be reserved for technical validation studies [1]. PWV can also be measured using non-invasive methods such as magnetic resonance imaging (MRI), commercially available devices, or even custom-built data acquisition systems (as seen in the Framingham and Asklepios population studies) [1]. Currently, there are several commercial devices that can automatically calculate regional or local PWV; however, most are used in research rather than clinical settings either due to their high costs or difficulty to operate [2]. One of these devices is the SphygmoCor device (AtCor Medical, Sydney, Australia), which has been used in more than 1400 peer-reviewed studies to noninvasively evaluate central arterial pressure waveforms and pulse wave velocities [1].

Another device is the PulsePen (DiaTecne, Milan, Italy), composed of a tonometer and an integrated electrocardiogram (ECG) unit that uses the ECG R-wave peak to determine PWV [2]. The Complior (Alam Medical, Colson, France), on the other hand, uses two piezoelectric mechanotransducers to measure the pressure pulses between two separated sites [2]. Another device called the Arteriograph (TensioMed, Budapest, Hungary) estimates PWV through oscillometric pressure curves based on plethysmography and pulsatile pressure changes [2]. These non-invasive devices include the external measurement of the wave pathway, which though desirable, also introduces a limitation due to the normal curvature of arterial pathways and the fact pulses do not travel along a direct path from the carotid to the femoral measuring site [1]. While catheterization is considered the gold standard to measure aortic PWV, its routine use as a potential diagnostic aid cannot be justified due to its extremely invasive nature; hence, commercial devices are most commonly employed [1].

Unfortunately, the routine use of PWV in the clinical setting has been hindered by the absence of established reference values. Since PWV seems to be affected by age, mean arterial pressure, sex, and conditions such as hypertension, it has been a challenge to establish a “normal” PWV range [4,5]. Several studies, however, have aimed to accomplish this goal among specific populations [4-7]. For example, a 2010 European study that examined 11,092 patients delivered a set of reference values based on sex, age, and blood pressure (BP) status [5]. They also found that pulse wave velocity increased both with increasing age and blood pressure levels in the reference value population [5]. Nevertheless, there are currently no widely accepted reference values for PWV for the various measurement methods.

Based on prior reports, it is known that arterial PWV is elevated in certain gastrointestinal conditions such as *Helicobacter pylori *(*H. pylori*) infection and inflammatory bowel disease (IBD) [8,9]. For example, *H. pylori *- a gram-negative bacterium that is transmitted primarily through the fecal-oral route and affects about two of three of the world’s population - has been implicated in the development of cardiovascular disease in some patients [8,10]. On the other hand, the relationship between PWV and inflammatory bowel disease (IBD) is one of the most studied due to the many extra-intestinal effects of IBD on the cardiovascular system [9,11,12]. Several studies focusing on the pathophysiology of IBD have found inflammatory cytokines, such as tumor necrosis factor-alpha (TNF-alpha), to play a fundamental role in the development and deposition of atherosclerotic plaque, which detrimentally affects the structure and functioning of endothelial tissues leading to coagulation issues and stiffening of blood vessels [9,12-14]. The chronic inflammation observed in IBD patients is thought to be responsible for the increase in PWV, thus affecting arterial stiffening, and prompting some researchers to use PWV as a way of measuring the efficacy of anti-TNF-alpha medications in reversing arterial wall stiffening [9]. In fact, a study published in 2019 successfully demonstrated that “a long‐term anti-TNF-alpha therapy in patients with IBD reduces aortic pulse‐wave velocity to a level comparable to that of healthy individuals” [9].

The purpose of this review is to clarify the effects of *H. pylori* infections and IBD on PWV and arterial wall stiffness. The goal is to highlight and explain the extent of the linkage between GI conditions and PWV, and to help evaluate the practicality of PWV as a potential clinical diagnostic aid.

## Review

In this review, published research articles discussing the relationship between two common gastrointestinal conditions and pulse wave velocity were evaluated. Emphasis was placed on pulse wave velocity calculation protocols, target population, results, and future implications.

Methods

**Figure 1:**
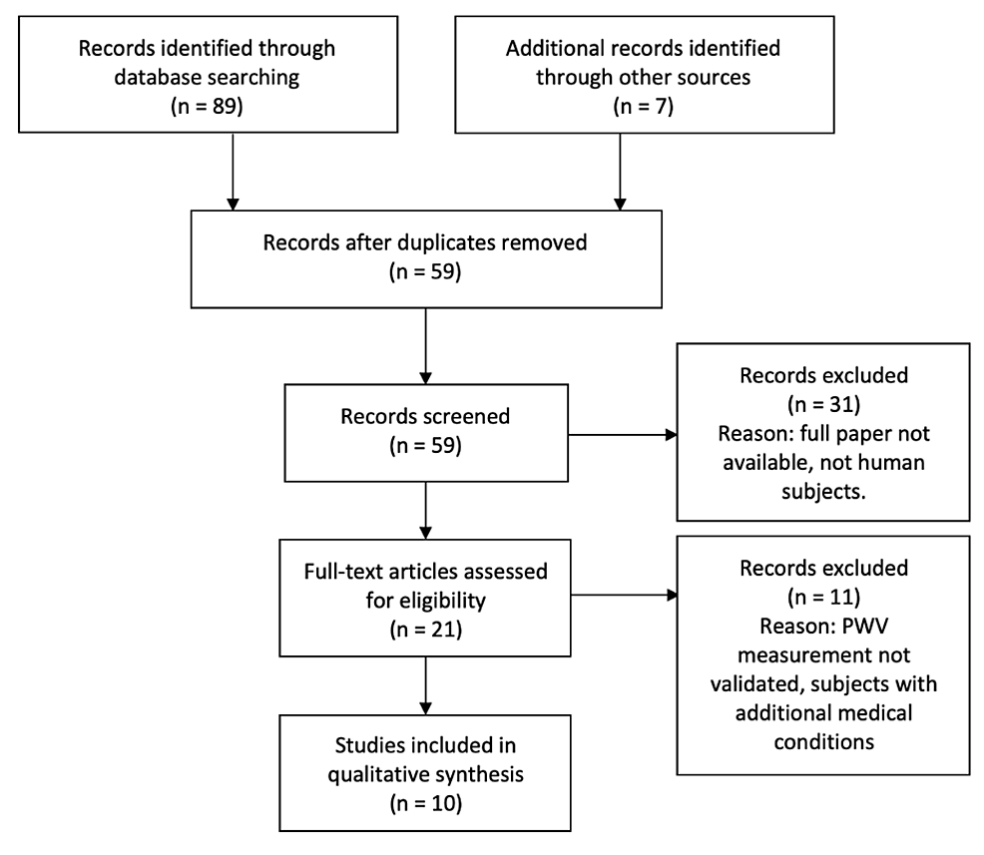
Search process flow chart

The following databases were used in this review: PubMed, CINAHL, EMBASE, Cochrane Central Register of Controlled Trials, and Biomedical Reference Collection: Comprehensive. English language articles were searched using search terms “pulse wave velocity” combined with either “*H. pylori*” or “IBD” present anywhere in the abstracts. Precedence was given to systematic reviews, meta-analyses, and clinical trials. Abstracts were examined for relevance and restricted to human subjects. The studies that investigated subjects with either *H. pylori* infections or IBD in the absence of any other gastrointestinal or major cardiovascular conditions were included; patients with hypertension or hyperlipidemia were also included. Pulse wave velocity measurement protocols were also screened to verify the use of validated methods as discussed in the introduction. Abstracts without full papers were excluded. Endnote X9 (Clarivate Analytics, Philadelphia, PA) was used to sort out all references and ensure seamless article citation. This review was conducted following the 2015 Preferred Reporting Item for Systematic Reviews and Meta-Analysis (PRISMA) guidelines with results shown in Figure 1. Databases were last accessed on March 20, 2021.

Results

A total of 89 results were obtained following a data search using keywords “pulse wave velocity” in combination with “*H. pylori*” or “IBD” present anywhere in the abstracts. After screening the abstracts using the inclusion criteria discussed in the Methods section of this review, a total of ten articles were included for analysis. 

Pulse Wave Velocity in H. pylori Infections

Gastritis, which indicates inflammation of the gastric mucosa, can be caused by infectious pathogens, most commonly *H. pylori *infections, dysregulation of the immune system, radiation, etc., or remain unknown [15,16]. Even though there is no universal classification system, and many controversies exist among physicians, it is commonly divided into acute, chronic, or special depending on its etiology, pathophysiology, and time course [15,17]. Most patients with *H. pylori* infections, however, will show features of both acute and chronic gastritis throughout the course of the infection [15]. Endoscopic and histopathological features are then essential to differentiate between the two [15]. 

Acute gastritis caused by *H. pylori* tends to be asymptomatic or very mild, although it may evolve into a chronic stage if not treated properly [18]. Acute histologic changes vary but can include neutrophilic infiltration in the lamina propria, pit abscesses, desquamation of the surface foveolar cells, and lymphoid follicles [18]. On the other hand, chronic gastritis due to *H. pylori* presents with signs and symptoms of peptic ulcer disease, gastric atrophy, intestinal metaplasia, and extra-gastrointestinal conditions such as iron deficiency anemia and idiopathic thrombocytopenic purpura [19]. Endoscopic examination for these patients, however, can appear normal in as many as 50% of the cases thus shifting the emphasis to histopathologic analysis for an accurate diagnosis [20].

A study published in 2003 investigated the role of *H. pylori *infection in the development of arteriosclerosis in several age groups [21]. The seroprevalence of *H. pylori* was established by measuring serum immunoglobulin G (IgG) antibody levels using an enzyme-linked immune-sorbent assay (ELISA). PWV was measured between the heart and carotid artery and between the heart and ankle. After adjusting for possible confounding values (age, sex, body mass index (BMI), smoking, and drinking habits), the heart-to-carotid artery PWV was found to be higher (p=0.027) in *H. pylori*-seropositive patients (n=49) younger than 39 when compared to seronegative individuals (n=107) of the same age group [21]. However, this trend tended to disappear with increased age and was not statistically significant for heart-to-carotid artery PWV [21]. These results indicate that *H. pylori* might increase arterial stiffness and affect the onset of cardiovascular disease in younger adults, whereas in older populations, aging, rather than infection with *H. pylori*, seems to play a bigger role in the increase of PWV and arterial stiffness [21].

A four-year follow-up study published in 2008 further investigated PWV in Japanese patients with *H. pylori* infections following a similar methodology [22]. However, while the unadjusted values for both heart-to-carotid artery PWV and heart-to-ankle PWV in *H. pylori*-seropositive subjects (n=166) tended to be higher than seronegative (n=92), after taking into account potential confounding variables, the adjusted values for PWV showed no significant difference in seronegative versus seropositive patients at the time of enrollment (p=0.436) or after four years (p=0.804) [22]. Furthermore, there was no difference in males vs. females and the percentage of change in PWV between the *H. pylori*-seropositive subjects and seronegative across age groups [22]. These results indicate that *H. pylori* seropositivity did not impact the age-related increase in PWV [22]. 

A 2005 research study also evaluated the relationship between *H. pylori* and arterial stiffness in 3412 male and 854 female Japanese subjects with no past medical history of coronary disease or stroke [23]. Subjects were also divided by sex and age groups (<=49 years old vs >=50 years old) with confounders such as age, BMI, medication for hyperlipidemia, hypertension, etc. taken into account [23]. Arterial stiffness was evaluated with PWV measurements from brachial-to-ankle using a volume-plethysmography device, while the anti-*H. pylori* antibody concentration was measured using ELISA [23]. A significant association was found between *H. pylori* seropositivity and increased PWV in male subjects younger than 49 (p=0.026) when compared to male subjects 50 or older (p=0.47) [23]. However, no significant association between *H. pylori* and PWV was observed in female subjects regardless of age group (p=0.58 for younger than 49 vs. p=0.86 for older than 50) [23]. As mentioned by the researchers, these results fail to justify a causal relationship between *H. pylori* infection and the development of arterial stiffness, as assessed by PWV [23].

A cross-sectional study published in 2018, however, found that *H. pylori* infections can be associated with subclinical but significant coronary artery stenosis in healthy populations, as assessed with cardiac multidetector computed tomography (MDCT), which evaluates the characteristics of intracoronary plaques [8]. Coronary artery calcium score (CACS) was also evaluated with the Agatston score, which is frequently used to assess the risk of coronary heart disease [24]. In this study, a diagnosis of* H. pylori* was established through esophagogastroduodenoscopy and the rapid urease test - also known as Campylobacter-like organism (CLO) test - which detects the presence of ammonia released by the enzymatic activity of urease (an enzyme released by *H. pylori*) [8,25]. For this study, the CACS was interpreted as having either no coronary calcium (CACS 0 vs. >0) or severe coronary calcium (CACS <=100 vs. >100) [8]. It was found that CACS >0 was significantly higher in the CLO-positive group (36.7% vs. 32.5%, p=0.05) than in the CLO-negative group, while the incidence of coronary stenosis was higher in the CLO-positive group (7.6% vs. 2.9%, p=0.01) [8]. However, there were no statically significant differences (p=0.79) in mean brachial-to-ankle PWV when comparing seropositive *H. pylori* patients to seronegative [8]. These findings indicate that while *H. pylori* might have some effect on the development of arterial stenosis, PWV failed to justify the results.

Pulse Wave Velocity in Inflammatory Bowel Disease

IBD is traditionally divided into two major pathologies - ulcerative colitis and Crohn’s disease [26]. Both disorders are characterized by unique risk factors, specific intestinal lesions, and clinical presentations. IBD has been known to cause extraintestinal manifestations in up to 40% of patients, and most commonly affects the skin and musculoskeletal system [26]. To a lesser extent, IBD has also been recognized as an important risk factor in the setting of cardiovascular disorders, including pericarditis, valvulopathies, venous and arterial thromboembolisms [26]. Studies focusing on the pathophysiology of IBD have found inflammatory mediators, such as cytokines, to play a fundamental role in the development and deposition of atherosclerotic plaque, which detrimentally affects the structure and function of endothelial tissues [14,26,27]. A cohort study of approximately 29,000 IBD patients showed a 1.6-fold increased risk for cardiovascular disease; however, major risk factors like smoking and obesity were not considered [14]. So far, the exact mechanism responsible for causing cardiovascular disease in IBD patients remains ambiguous.

In 2012, Zanoli et al. concluded that “arterial stiffness is increased in patients with inflammatory bowel disease independent of conventional cardiovascular risk factors” [13]. In this study, a diagnosis of IBD was established through clinical, laboratory, endoscopic, and histological criteria while the carotid-to-femoral artery PWV was measured using the SphygmoCor device [13]. Subjects included in this study were between the ages of 19 years and 49 years with no cardiovascular risk factors, including hypertension, hyperlipidemia, and diabetes mellitus [13]. Overall, it was found that carotid-to-femoral artery PWV was higher in the 32 IBD patients compared to the 32 healthy controls (6.6 +/- 1.4 vs. 6.0 +/- 0.8 m/s, p<0.05) [13]. This group also measured the carotid-to-radial artery PWV and reported that it correlated with disease duration in IBD patients (+0.11 m/s per one year of aging) and was higher in patients with IBD vs. controls (8.5 vs 7.2 m/s, p<0.001) [13]. They found no significant differences in either of these PWVs between patients with ulcerative colitis or Crohn’s disease [13]. Contrastingly, a study published in 2014, using similar IBD diagnosis criteria and PWV calculations as Zanoli et al. yielded different results [14]. They compared 44 IBD patients, ages 18 to 60 years old with no history of cardiovascular disease, with 44 healthy controls and found no statistically significant difference in PWV between them (6.8 ± 1.2 m/s vs. 6.4 ± 0.9 m/s) [14]. However, results showed that carotid-to-femoral PWV was higher in patients with Crohn’s disease vs. patients with ulcerative colitis (7.0 ± 1.2 vs 6.3 ± 1.2 m/s; p=0.044) [14]. This study, overall, showed no changes in arterial stiffness, as assessed by PWV, in patients with IBD [14].

A 2015 cross-sectional study used the arteriography device to record carotid-to-femoral PWV and evaluate arterial stiffness in patients with IBD and no cardiovascular risk factors like hypertension, diabetes, and hyperlipidemia [27]. They reported that carotid-to-femoral PWV was higher in patients with Crohn’s disease (n=52) and ulcerative colitis (n=74) vs. healthy controls (n=66) (8.16 m/s, 8.13 m/s, 6.85 m/s, p<0.001), respectively [27]. No significant differences in PWV were found between Crohn’s and ulcerative colitis patients but carotid-to-femoral artery PWV was correlated with disease duration (p=0.001) [27]. These results suggest that patients with IBD, in the absence of cardiovascular disease, are at an increased risk for arterial stiffening [27].

In 2019, Zanoli et al. carried out a multicenter, longitudinal study to test the hypothesis that chronic inflammation in IBD patients was responsible for the increase in PWV and to evaluate if anti-TNF-alpha medications could reduce PWV in these patients [9]. In this study, IBD diagnosis was established through clinical and endoscopic evaluation and carotid-to-femoral artery PWV was determined using the SphygmoCor device [9]. The study included 82 patients with ulcerative colitis, 85 patients with Crohn’s disease, and 167 healthy controls [9]. Pre-treatment baseline values of PWV for ulcerative colitis and Crohn’s were greater (7.8 m/s, 7.9 m/s) respectively when compared to healthy controls (7.1 m/s, p<0.001) [9]. After following these patients for a median of four years, they found that patients treated with anti-TNF-alpha therapy experienced an aortic de-stiffening (8.5 m/s at baseline vs. 7.9 m/s at follow-up, p=0.02) when compared to IBD patients treated with another therapy like salicylates [9]. These results suggest that effectively controlling inflammation may reduce cardiovascular risk factors in patients with IBD [9].

A cross-sectional observational study in 2020, following similar IBD diagnosis criteria and PWV calculations as Zanoli et al., concluded that carotid-to-femoral artery PWV was also higher in IBD patients when compared to healthy controls (8.06 ± 3.23 vs. 6.42 ± 1.47 m/s, p<0.001) [28]. However, they found no significant association between disease duration and PWV as well no significant difference between IBD groups [28]. Interestingly, they also found no difference in arterial stiffness between IBD subgroups based on the type of therapy, which included monoclonal antibody biologics (which mimic the immune system response and include drugs that target TNF-alpha, e.g., infliximab), aminosalicylates (e.g., sulfasalazine, olsalazine), and disease-modifying antirheumatic drugs (DMARD, e.g. methotrexate, hydroxychloroquine) [28-30].

Inflammatory bowel disease, although rare in children, can present as early as infancy, while 5-10% of patients develop IBD during childhood or adolescence [31,32]. At presentation, children are also more likely to have extensive intestinal involvement and rapid progression when compared to adults [33,34]. A pilot study published in 2017, claimed to be the first one to use carotid-to-femoral artery PWV to examine the extent of arterial stiffness in children with IBD [35]. Results showed that all 25 children evaluated had a normal carotid-to-femoral PWV with no difference between ulcerative colitis and Crohn’s disease patients (4.4 m/s vs. 4.6 m/s, p=0.4) [35]. Additionally, there was no correlation between PWV and disease duration, although it is noteworthy to mention that majority of the children (68%) were in clinical remission, indicated by the pediatric ulcerative colitis activity index (PUCAI) used for children with ulcerative colitis and the Pediatric Crohn’s Disease Activity Index (PCDAI) used for children with Chron’s disease (PUCAI/PCDAI scores <10) [35].

Discussion

While some studies have claimed a causal relationship between *H. pylori* infections and the onset of cardiovascular disease, some of the papers examined in this review have shown mixed results [8,22]. When examining the unadjusted PWV data of many of these articles, PWV was seen to increase in *H. pylori*-seropositive patients when compared to seronegative patients across all age groups [8,22,23]. However, after accounting for confounders such as age, body mass index, sex, smoking, and alcohol history, significant changes were only seen in one study for younger patients while no substantial changes were observed in older patients [21]. Furthermore, another study showed no differences between *H. pylori*-seropositive male and female patients [22]. The diagnostic method for *H. pylori* infections also varied. Some of the studies used serologic testing as means of diagnosis while others used the more invasive CLO test [14,22]. This difference is noteworthy since there have been concerns about the accuracy of serologic tests due to their lower overall sensitivity (85%) and specificity (79%) when compared to other diagnostic tests [25]. However, the more invasive nature of the CLO test limits its use in the research setting.

Due to the chronic inflammation observed in patients with inflammatory bowel disease, researchers have hypothesized that pulse wave velocity, as a measurement of arterial stiffness, will be increased in these patients. While most of the studies analyzed in this review agreed that PWV is indeed increased in IBD and that no significant changes in PWV exist between ulcerative colitis and Crohn’s disease, one study concluded the opposite [14]. After comparing the methodologies and population screening protocols for the studies examined in this review, it was determined they followed very similar protocols while ending with different results. However, the consensus seems to be that carotid-femoral pulse wave velocity increases in patients older than 18 years with inflammatory bowel disease for both sexes [9,13].

Another important result observed in one of the studies was that carotid-to-radial artery PWV, rather than carotid-to-femoral PWV, could be correlated with disease duration in IBD patients but no correlation was established with the severity of IBD [13]. In fact, it is noteworthy to highlight that 88% of the IBD patients were in remission - usually indicated by the absence of gastrointestinal symptoms and changes in biomarker levels, although not explained in this study - while only 12% had active disease [13,36]. Lastly, one study showed that carotid-femoral artery PWV decreased in patients with IBD who were treated with anti-TNF alpha therapy while those on salicylates experienced an increase in aortic stiffening [9]. These investigators concluded that PWV was reduced to a level similar to that of controls after treatment with anti-TNF alpha therapy suggesting that effective control of inflammation may reduce the degree of arterial stiffness by normalizing PWV, which has been previously established as an indicator of cardiovascular disease risk [1,9].

Some of the inconsistent results observed in the studies reviewed here might be due to the different methods used to determine PWV. While all the studies included external measurements for the pulse pathway, they varied by which two vessels of the arterial tree were used and which devices were employed. For example, one of the studies that evaluated patients with *H. pylori* infection used heart-to-carotid PWV and heart-to-brachial artery PWV while another, similar study used brachial-ankle PWV [22,23]. This change might have caused discrepancies in the results since heart-to carotid artery PWV is not widely used and its clinical significance has not been fully established [37]. Lastly, since inflammatory bowel does not present equally in all patients, test subjects were also divided into active disease and remission, without further explanation as to what they considered “remission” or “active” disease, potentially affecting the results [13].

## Conclusions

In this review, the association between PWV and two common gastrointestinal conditions was evaluated. Based on the reported evidence it appears that *H. pylori* infections might play an important role in the development of arterial stiffening in the young, whereas in older populations, the effect of *H. pylori* on PWV appears to be minimal. While some studies have concluded that *H. pylori* infections can be linked with the incidence of atherosclerosis, PWV does not appear to be the best parameter to assess the risk of developing cardiovascular disease in these older patients. On the other hand, PWV has been frequently shown to increase in patients with IBD suggesting that PWV might be a useful parameter to estimate arterial stiffness in these patients, and thus the risk of developing future cardiovascular disease. PWV could also be potentially used to assess the efficacy of anti-TNF-alpha immunotherapy on arterial wall stiffness, and thus predict the risk of developing cardiovascular manifestations in patients with inflammatory bowel disease.

Cardiovascular disease affects numerous people from across all age groups and ethnicities. It has been implicated in numerous medical conditions and recognized as an important mortality risk factor. Although pulse wave velocity, as a parameter for arterial stiffness was not significantly changed in patients with *H. pylori*, it is notable to mention that signs of atherosclerosis were in fact observed through the assessment of other parameters, for example, coronary artery calcium score and degree of coronary artery stenosis. This is a clear indication that more studies are needed to evaluate the relationship between *H. pylori* and its causal effect on cardiovascular disease. On the other hand, pulse wave velocity has been demonstrated as an accurate parameter to evaluate arterial stiffness in patients with inflammatory bowel disease. These findings could be potentially extrapolated to other immune-related diseases, such as systemic lupus, where exposure to chronic inflammation might cause arterial wall changes. PWV, for example, could be used to assess the impact of immunotherapy on arterial stiffening in these patients and their risk to develop adverse cardiovascular events.
